# A comprehensive description of the TolC effect on the antimicrobial susceptibility profile in *Enterobacter bugandensis*

**DOI:** 10.3389/fcimb.2022.1036933

**Published:** 2022-12-09

**Authors:** Bingyu Li, Ji Zhang, Xiaodong Li

**Affiliations:** ^1^ Health Science Center, Shenzhen University, Shenzhen, Guangdong, China; ^2^ Key Laboratory of Livestock Infectious Diseases in Northeast China, Ministry of Education, Key Laboratory of Zoonosis, Shenyang Agricultural University, Shenyang, China; ^3^ Research and Development Center, Panjin Guanghe Crab Industry Co., Ltd., Panjin, China

**Keywords:** TolC, efflux pumps, antimicrobial agents, susceptibility, synergistic effects, *Enterobacter bugandensis*

## Abstract

**Background:**

Enterobacter bugandensis is an emerging human pathogen in which multidrug resistant strains have been continuously isolated from various environments. Thus, this organism possesses the potential to pose challenges in human healthcare. However, the mechanisms, especially the efflux pumps, responsible for the multidrug resistance in E. bugandensis remain to be well elucidated.

**Methods:**

The Enterobacter strain CMCC(B) 45301 was specifically identified using whole genome sequencing. The specific CMCC(B) 45301 homologues of the TolC dependent efflux-pump genes characterized in Escherichia coli were identified. The tolC deletion mutant in CMCC(B) 45301 was constructed and subjected to susceptibility tests using 26 different antimicrobial agents, along with the wild type strain. The synergistic effects combining the Bacillus crude extract (BCE) and several other TolC-affected compounds against CMCC(B) 45301 were assayed.

**Results:**

We reclassified the Enterobacter CMCC(B) 45301 strain from species cloacae to bugandensis, on the basis of its whole genome sequence. We found that the CMCC(B) 45301 TolC, AcrAB, AcrD, AcrEF, MdtABC, EmrAB, and MacAB exhibit high similarity with their respective homologues in E. coli and Enterobacter cloacae. Our results for the susceptibility tests revealed that lacking tolC causes 4- to 256-fold decrease in the minimal inhibitory concentrations of piperacillin, gentamicin, kanamycin, tetracycline, norfloxacin, ciprofloxacin, chloramphenicol, and erythromycin against CMCC(B) 45301. In addition, the inhibition zones formed by cefuroxime, cefoperazone, amikacin, streptomycin, minocycline, doxycycline, levofloxacin, florfenicol, trimethoprim-sulfamethoxazole, azithromycin, lincomycin, and clindamycin for the tolC mutant were larger or more obvious than that for the parent. Our data suggested the important role played by TolC in CMCC(B) 45301 susceptibility to common antibiotic families covering ß-lactam, aminoglycoside, tetracycline, fluoroquinolone, phenicol, folate pathway antagonist, macrolide, and lincosamide. Deletion for tolC also increased the susceptibility of CMCC(B) 45301 to berberine hydrochloride and BCE, two natural product-based agents. Finally, we found that erythromycin, norfloxacin, and ciprofloxacin can potentiate the antibacterial activity of BCE against CMCC(B) 45301.

**Discussion:**

The present study elaborated the comprehensive TolC effect on the antimicrobial susceptibility profile in E. bugandensis, which might contribute to the development of more therapeutic options against this nosocomial pathogen.

## Introduction

*Enterobacter* spp. are gram-negative bacteria inhabiting a wide range of environmental niches with the human gastrointestinal tract being the most noticeable one (Mezzatesta et al., 2012). Some members of genus *Enterobacter* are well-known nosocomial pathogens, capable of causing infections in the blood streams, lungs, urinary tracts, and peritoneum of immunocompromised individuals, especially those accepting intensive cares in hospitals (Chow et al., 1991; Fernandez-Baca et al., 2001; Davin-Regli and Pages, 2015; Gravey et al., 2020). In the past decades, clinical isolates possessing multidrug resistance resulted from production of extended spectrum ß-lactamases (ESBLs) and carbapenemases, overexpression of AmpC, reduction in membrane permeability, and overexpression of efflux pumps are frequently isolated in *Enterobacter*, which poses a tremendous challenge to human healthcare, world-widely (Sanders and Sanders, 1997; Hilty et al., 2013; Chavda et al., 2016; Telke et al., 2017; Liu et al., 2021). *Enterobacter bugandensis* was identified as a novel species by Doijad and colleagues (Doijad et al., 2016), and subsequently expanded as the identification of strains isolated from the international space station (ISS), various nosocomial settings, and vegetables (Singh et al., 2018; Falgenhauer et al., 2019; Matteoli et al., 2020; Moon et al., 2021). As known, conventional typing methods based on 16S rDNA sequencing and phenotypic assays are not adequate for accurate identification of species within genus *Enterobacter* (Annavajhala et al., 2019). Recently, as the growing use of whole genome sequencing, the resolution of molecular typing of bacterial strains has been elevated to the genome level (Federhen et al., 2016; Ciufo et al., 2018). This facilitated the reclassification of some strains from *Enterobacter cloacae* to *E. bugandensis*, which was based on the average nucleotide identity (ANI) as compared their respective genomes against known *E. bugandensis* strains (Ciufo et al., 2018; Matteoli et al., 2020). Notably, the first identified *E. bugandensis* strains were neonatal-blood isolates, indicating their potential causing severe systemic infections in human beings (Doijad et al., 2016). The pathogenicity of *E. bugandensis* was evidenced by the results that *E. bugandensis* EB-247 establishes faster infections in *Galleria mellonella* and yields a higher survival rate grown in human serum, compared with *E. cloacae* ATCC 13047, a known pathogenic strain of *Enterobacter* (Pati et al., 2018). Thus, *E. bugandensis* has been considered as the most pathogenic species within genus *Enterobacter* (Pati et al., 2018; Matteoli et al., 2020). Besides, the multidrug resistant (MDR) strains of *E. bugandensis* have been continuously isolated (Doijad et al., 2016; Moon et al., 2021). Taken together, reclassification or specific identification of the *E. bugandensis* strains is exceedingly required, in order to provide references important for precise reaction combating this organism and effective genetic engineering for better understanding of this species.

Efflux pumps are important mechanisms for the emergence of multidrug resistance in gram-negative bacteria and comprise classes of RND, MF, ABC, MATE, SMR, and PACE, see reference Nikaido and Zgurskaya, 1999; Li et al., 2015 and Nishino et al., 2021 for reviews. The RND transporters are tripartite efflux pumps, among which AcrAB-TolC is the major one responsible for expelling many antimicrobial agents in *Enterobacter* (Perez et al., 2007; Guerin et al., 2016). It has been described that substrates of AcrAB-TolC encompass almost all the commonly-used antibiotic families, namely ß-lactam inhibitor, aminoglycoside, tetracycline, macrolide, lincosamide, fluoroquinolone, folate pathway antagonist, and phenicol, in some *Enterobacter* strains (Perez et al., 2007; Guerin et al., 2016; Liu et al., 2018; Gravey et al., 2020). Alarmingly, AcrAB-TolC can confer, at least in part, the increased resistance to some effective pharmaceutical options against MDR strains, like the combination of ß-lactam/ß-lactamase inhibitor and the last-line drugs, carbapenems, polymyxins, and tigecycline, in *Enterobacter* spp. (Perez et al., 2007; Telke et al., 2017; Huang et al., 2019; Gravey et al., 2020; Senchyna et al., 2021). Efflux of antimicrobial natural products can also be performed by AcrAB-TolC in *Enterobacter* strains (Kuete et al., 2010). Furthermore, *Enterobacter* strains are able to gain spontaneous mutations in the *acrB* gene or genes encoding regulators of *acrAB* and *tolC*, for their adaptations to antibiotic stresses (Telke et al., 2017; Gravey et al., 2020; Senchyna et al., 2021). As an outer-membrane channel, TolC can form efflux pumps with some other RND-type transporters, MF transporters, and ABC transporters, overexpression of which is capable of restoring the resistance to one or more antibiotic agents in bacterial strains devoid of AcrB (Kobayashi et al., 2001; Nagakubo et al., 2002; Nishino et al., 2003; Li et al., 2015; Yousefian et al., 2021; Nishino et al., 2021). Thus, studies regarding the TolC-involved efflux pumps in gram-negative bacteria including *Enterobacter* spp. are imperative for developing novel therapeutic strategies, for instance, the combination of efflux-pumped antibiotic/efflux-pump inhibitor, against the MDR strains (Morita et al., 2016; Li et al., 2017; Grimsey et al., 2020; Li et al., 2021b).

In *E. bugandensis*, some isolates are tested positive for the resistance/reduced susceptibility to cephalosporins, imipenem, aminoglycosides, fluoroquinolones, and polymyxin B (Doijad et al., 2016; Moon et al., 2021; Sarangi et al., 2022). Studies revealed that the plasmid-borne resistance genes and the chromosomally integrated gene encoding imipenemase IMI-1 might contribute to the multidrug resistance in *E. bugandensis* EB-247 and the imipenem resistance in *E. bugandensis* S68-1, respectively (Pati et al., 2018; Moon et al., 2021). However, investigations of the multidrug resistance/susceptibility concerning efflux pumps in *E. bugandensis* are still lacking. To our knowledge, a comprehensive study about the correlation between TolC or TolC-related efflux pumps and multidrug resistance/susceptibility in *E. bugandensis* has not been reported. Here, we constructed a clean deletion mutant of *tolC* in the *E. bugandensis* CMCC(B) 45301 strain, and tested the changes in its susceptibility to a range of 26 antimicrobial agents, including antibiotics belonging to different families and natural products, as compared with the parent strain. In addition, certain combinations of the TolC-affected antibiotic and natural product were assessed for their synergistic effects against the wild-type strain, for exploring novel antibacterial options combating *E. bugandensis*. The present study mainly aimed to elucidate the effect of TolC on the antimicrobial susceptibility profile in *E. bugandensis*, in advance providing information assisting actions against this bacterium in the future.

## Results

### Whole genome sequencing-based reclassification of *E. cloacae* CMCC(B) 45301

In this study, we used *E. cloacae* CMCC(B) 45301, a strain collected in China National Center for Medical Culture Collections (CMCC; http://www.cmccb.org.cn/cmccbnew/) and initially identified as *E. cloacae*, as the experimental material. To obtain the comprehensive genetic information of this strain, we sequenced and assembled its complete genome using the combination of Pacbio and Illumina platforms, see materials and methods. Our results showed that the circular chromosome DNA of CMCC(B) 45301 (GenBank accession No. CP097255) possesses a size of 4,631,472 base pairs (bp) and a G+C content of 56.13%. In addition, strain CMCC(B) 45301 harbors an 81,691-bp plasmid (GenBank accession No. CP097254) with a G+C content of 47.35%. The chromosome and plasmid sequences contain a total of 4,499 genes annotated *via* the NCBI Prokaryotic Genome Annotation Pipeline (PGAP). Importantly, according to the result of the quality-control test that compared the genome sequence of CMCC(B) 45301 against the type-strain genomes throughout GenBank as previously described (Federhen et al., 2016), strain CMCC(B) 45301 should be identified more specifically as an *E. bugandensis* organism, given the 98.8% ANI comparing its genomic assembly (GenBank accession No. GCA_023374275.1) with the best-matching type-strain assembly (GenBank accession No. GCA_019046905.1) of *E. bugandensis* (Ciufo et al., 2018). Thus, the original *E. cloacae* CMCC(B) 45301 strain was reidentified and designated as *E. bugandensis* CMCC(B) 45301 (EBU45301).

### Identification of the TolC dependent efflux-pump genes in EBU45301

As known, TolC can form efflux pumps with the RND-type transporters, AcrAB, AcrAD, AcrEF, MdtEF, and MdtABC, the MF-type transporters, EmrAB and EmrKY, and the ABC-type transporter MacAB in *Escherichia coli* (Nishino et al., 2003). Recently, effects of *acrD*, *acrEF*, *mdtABC*, and some other RND efflux-pump genes on the resistance/susceptibility to various antibiotics in the *E. cloacae* ATCC 13047 strain (ECL13047) were characterized, describing a landscape of TolC-dependent RND efflux pumps in *E. cloacae* highly similar to that in *E. coli* (Guerin et al., 2016). To locate the respective EBU45301 homologues of the TolC dependent efflux-pump genes in *E. coli* K-12 MG1655 (GenBank accession No. U00096.3), BLAST was performed comparing each of these *E. coli* genes against the whole genome of EBU45301, and the locus whose product displayed distinguishable-high identities/positives with that of the corresponding *E. coli* gene was considered as a specific homologue. The identified EBU45301 efflux-pump genes were also subjected to comparisons with their respective homologues in ECL13047 (GenBank accession No. NC_014121), for further confirmation. Our results revealed that specific homologues of *tolC*, *acrAB*, *acrD*, *acrEF*, *mdtABC*, *emrAB*, and *macAB* are encoded in both EBU45301 and ECL13047, and yet such homologues for *mdtEF* and *emrKY* can be found throughout the genome of neither of these strains (**Table 1**). EBU45301 harbors genes encoding for some other RND efflux-pump components, whose specific homologues are either present or absent in ECL13047 (**Tables S1**, **S2**), exhibiting potential in interaction with TolC (Guerin et al., 2016). In addition, several MF efflux pump proteins showing partial homology with the respective EmrAB- and EmrKY-TolC components were identified in EBU45301 (**Table S3**), indicating that there are EmrAB/EmrKY-like efflux pumps involving TolC in this bacterium.

**Table 1:** TolC related efflux-pump genes in EBU45301 located based on their homologies with the respective sequences in *E. cloacae* and *E. coli*.

Locus tag EBU45301	Locus tag ECL13047^a^	Gene *E. coli*	Identities/positives (%)^b^ EBU45301 *versus* (vs.) ECL13047	Identities/positives (%) EBU45301 vs. *E. coli*
RND type				
M1V99_19125	ECL_RS21755	*tolC*	96/98	85/92
M1V99_05805	ECL_RS06015	*acrA*	95/97	87/93
M1V99_05800	ECL_RS06010	*acrB*	99/99	93/96
M1V99_16450	ECL_RS18730	*acrD*	97/98	91/96
M1V99_20240	ECL_RS23185	*acrE*	94/96	75/86
M1V99_20245	ECL_RS23190	*acrF*	96/98	84/92
ND^c^	ND	*mdtE*	NA^c^	NA
ND	ND	*mdtF*	NA	NA
M1V99_15070	ECL_RS16825	*mdtA*	96/98	85/91
M1V99_15075	ECL_RS16830	*mdtB*	97/99	90/96
M1V99_15080	ECL_RS16835	*mdtC*	98/99	92/97
MF type				
M1V99_17535	ECL_RS20065	*emrA*	98/99	86/93
M1V99_17540	ECL_RS20070	*emrB*	99/100	93/98
ND	ND	*emrK*	NA	NA
ND	ND	*emrY*	NA	NA
ABC type				
M1V99_07880	ECL_RS13615	*macA*	97/98	83/91
M1V99_07885	ECL_RS13610	*macB*	97/98	87/92

^a^ Genes in ECL13047 has been designated with new locus tags, which are different from the ones described in reference Guerin et al., 2016.

^b^ This result reveals the similarity of an EBU45301 amino acid sequence against its respective homologues in ECL13047 and *E. coli* K-12 MG1655. The query covers (%) of sequences used in alignments for each of the EBU45301 protein are all larger or equal to 98.

^c^ ND, non-detectable; NA, non-applicable.

**Figure 1:**
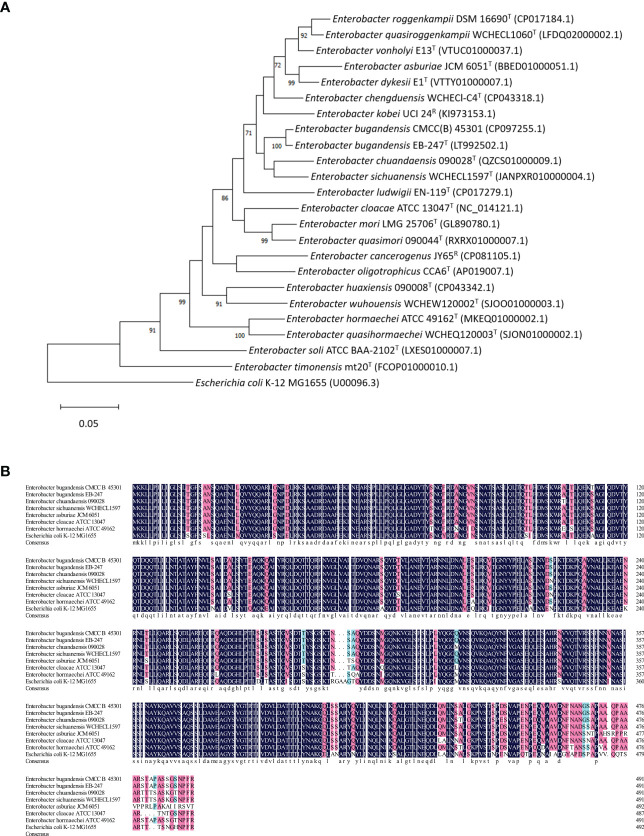
Comparative analyses of EBU45301 and the type/reference strains representing *Enterobacter* species based on the *tolC* gene. **(A)** A maximum-likelihood tree was constructed based on the *tolC* gene sequences retrieved from the genomes of the *Enterobacter* strains as indicated. The Genbank accession No. for each of the used genomic sequence was shown in parentheses. The shown values on certain branch nodes were bootstrap values >70%, based on 500 replications. The bar represents 0.05 substitutions per site. *E. coli* K-12 MG1655 was used to root the tree. T, type; R, reference. **(B)** An alignment of the amino acid sequences of TolC from EBU45301 and several other *Enterobacter* strains (as indicated) was performed. The homology levels were shaded as follows: ≥50%, light blue; ≥70%, pink; =100%, dark blue. The consensus sequence was shown at the bottom of this alignment in lower case.

We also compared the *tolC* gene of EBU45301 with that of the type or recommended reference (by the NCBI Genome database) strains representing the species belonging to genus *Enterobacter*. A maximum-likelihood tree was constructed based on *tolC*, and it is shown that EBU45301 is clustered with *E. bugandensis* EB-247 in the same clade closely related to *Enterobacter chuandaensis* 090028 and *Enterobacter sichuanensis* WCHECL1597 (**Figure 1A**). Furthermore, the TolC amino-acid sequences from EBU45301, *E. bugandensis* EB-247, *Enterobacter chuandaensis* 090028, *Enterobacter sichuanensis* WCHECL1597, *Enterobacter cloacae* ATCC 13047, *Enterobacter asburiae* JCM 6051, *Enterobacter hormaechei* ATCC 49162, and *E. coli* K-12 MG1655 were aligned for a comparison, to investigate the homology of EBU45301 TolC with its respective homologues in the closely related species and species in which AcrAB-TolC has been reported as a powerful MDR mechanism (Li et al., 2015; Guerin et al., 2016; Telke et al., 2017; Gravey et al., 2020). The alignment result revealed that EBU45301 TolC is highly homologous with the other TolC sequences (**Figure 1B**), suggesting its function transporting multiple compounds. Taken together, we hypothesized that TolC plays a magnificent role in mediating the antimicrobial susceptibility profile of EBU45301.

### Investigation of the TolC effect on the susceptibility of EBU45301 to different antimicrobial agents

To study the role of TolC in EBU45301 physiology, we constructed a clean deletion mutant for *tolC via* the pRE112 system (Edwards et al., 1998). It has been described that the TolC-dependent efflux pumps affect the susceptibility of *E. coli* and some *Enterobacter* species to a broad spectrum of antibiotics (see **Table S4**) and natural products like isobavachalcone and Diospyrone (Kuete et al., 2010). Here, we assayed the susceptibility of the *tolC* mutant (*tolC*) and the wild-type strain (WT) to a range of 26 antimicrobial agents, covering the extensively-applied antibiotic families, ß-lactam, aminoglycoside, tetracycline, fluoroquinolone, macrolide, lincosamide, phenicol, and folate pathway antagonist, and two other agents, berberine hydrochloride (BBH) and the *Bacillus* crude extract (BCE), see materials and methods for details.

Our results showed that the wild-type EBU45301 is intrinsically resistant to cefazolin and susceptible to piperacillin, cefuroxime, ceftazidime, ceftriaxone, cefoperazone, imipenem, amikacin, gentamicin, kanamycin, streptomycin, tetracycline, minocycline, doxycycline, norfloxacin, ciprofloxacin, levofloxacin, chloramphenicol, and trimethoprim-sulfamethoxazole (**Tables 2**–**4**), according to the respective breakpoints recommended in the performance standards provided by CLSI, 2022. We also found that the *tolC* deletion causes the increased susceptibility of EBU45301 to all the tested antimicrobial agents, except for cefazolin, ceftazidime, ceftriaxone, and imipenem (**Tables 3**, **4**). The minimal inhibitory concentration (MIC) of piperacillin for the *tolC* mutant was 8-16 times smaller than that for the parent (**Table 3**), in accordance with the disk-diffusion results for piperacillin (**Table 4** and **Figure S1**). In terms of drugs classified as cephalosporins, the *tolC* deletion rendered EBU45301 more sensitive to cefuroxime and cefoperazone, different from that for cefazolin, ceftazidime, and ceftriaxone (**Tables 3**, **4**, and **Figure S1**). In addition, loss of *tolC* resulted in a 4-fold decrease in the MICs of gentamicin, kanamycin, and tetracycline against EBU45301 (**Table 3**), which was consistent with the slightly larger inhibition zones for the *tolC* mutant than WT, revealed in the disk-diffusion tests for these drugs (**Table 4** and **Figure S1**). Similarly, in the cases of the other two aminoglycosides, amikacin and streptomycin, the effect of TolC was observed but not very obvious (**Table 4** and **Figure S1**). However, not like that for tetracycline, strain *tolC* appeared to be much more sensitive to minocycline and doxycycline, also belonging to family tetracycline, than WT, which might be due to the weaker activity of these two antibiotics against the wild-type EBU45301 (**Table 4** and **Figure S1**). The TolC effect was found remarkable on the tested fluoroquinolones. The respective fold changes in MIC for norfloxacin and ciprofloxacin were 64 and 32, comparing *tolC* with WT (**Table 3**). These phenotypes in susceptibility observed using the broth-microdilution method were confirmed *via* the well-diffusion method, for norfloxacin and ciprofloxacin (**Table 4** and **Figure S1**). Moreover, the inhibition zone using levofloxacin for strain *tolC* was obviously larger than strain WT, suggesting a TolC effect on levofloxacin efflux as strong as that for norfloxacin or ciprofloxacin (**Table 4** and **Figure S1**). Moreover, compared with the wild type, the *tolC* mutant exhibited defects in resisting chloramphenicol and florfenicol, which were determined as the 8-fold decrease in MIC (**Table 3**) and the much larger inhibition zone as shown (**Table 4** and **Figure S2**), respectively. Trimethoprim-sulfamethoxazole, a combination of folate pathway antagonists, showed higher activity combating *tolC* than WT, assayed through the disk-diffusion tests (**Table 4** and **Figure S2**). We also tested the TolC effect on EBU45301 susceptibility to antibiotics that are not recommended for cases of *Enterobacteriaceae*, such as macrolides and lincosamides. Larger and clearer inhibition zones were observed for the *tolC* mutant than the WT strain, when the susceptibility to erythromycin, azithromycin, and clindamycin was tested (**Table 4** and **Figure S2**). In assays using lincomycin, there were areas with fewer bacteria instead of clear zones around the disks for *tolC*, and yet none of these were found for WT (**Table 4** and **Figure S2**). Note that there is a 256-fold decrease in the MIC of erythromycin against *tolC*, compared with WT (**Table 3**), indicating that the efflux of erythromycin is tightly related to TolC in EBU45301.

**Table 2:** Antibiotic susceptibility profile of the wild-type EBU45301.

Antibiotic	Susceptibility	MIC (mg/L^a^)	Diameter (mm^a^)
ß-lactam			
Piperacillin	Susceptible	≤8	
Cefazolin	Resistant	≥8	
Cefuroxime	Susceptible		≥18
Ceftazidime	Susceptible	≤4	
Ceftriaxone	Susceptible		≥23
Cefoperazone	Susceptible		≥21
Imipenem	Susceptible	≤1	
Aminoglycoside			
Amikacin	Susceptible		≥17
Gentamicin	Susceptible	≤4	
Kanamycin	Susceptible	≤16	
Streptomycin	Susceptible		≥15
Tetracycline			
Tetracycline	Susceptible	≤4	
Minocycline	Susceptible		≥16
Doxycycline	Susceptible		≥14
Fluoroquinolone			
Norfloxacin	Susceptible	≤4	
Ciprofloxacin	Susceptible	≤0.25	
Levofloxacin	Susceptible		≥21
Phenicol			
Chloramphenicol	Susceptible	≤8	
Folate pathway antagonist			
Trimethoprim-sulfamethoxazole	Susceptible		≥16

^a^ mg/L, milligram/liter; mm, millimeter.

**Table 3:** Phenotypes of the EBU45301 *tolC* mutant in drug susceptibility (microdilution method).

Antimicrobial	MIC (mg/L) for			
	WT	*tolC*	*tolC*/complementation^a^	*tolC*/vector^b^
ß-lactam				
Piperacillin	2-4	0.25	2	0.25
Cefazolin	64	64		
Ceftazidime	0.5	0.5		
Imipenem	0.125	0.125		
Aminoglycoside				
Gentamicin	2	0.5	1	0.5
Kanamycin	2	0.5	1	0.25-0.5
Tetracycline				
Tetracycline	2	0.5	2	0.5
Fluoroquinolone				
Norfloxacin	0.384	0.006	0.192	0.006
Ciprofloxacin	0.032	0.001	0.016	<0.001
Phenicol				
Chloramphenicol	8	1	8	1
Macrolide				
Erythromycin	1,536	6	768	6
Natural product				
BBH^c^	>1,024	512	>1,024	512
BCE^d^	0.031x original	0.016x original	0.031x original	0.016x original

^a^ For tests with tetracycline, BBH, and BCE, the *tolC*/complementation strain used was *tolC*/pBYL030. For tests with chloramphenicol, the *tolC*/complementation strain used was *tolC*/pBYL032. For the rest cases, the *tolC*/complementation strain used was *tolC*/pBYL024.

^b^ For tests with tetracycline, BBH, and BCE, the *tolC*/vector strain used was *tolC*/pBYL031. For tests with chloramphenicol, the *tolC*/vector strain used was *tolC*/pBYL033. For the rest cases, the *tolC*/vector strain used was *tolC*/pACYC184.

^c^ The same amount of DMSO as that contained in 2,048 mg/L BBH could completely inhibit the growth of EBU45301. Thus, the activity of 2,048 mg/L BBH against WT could not be assessed. For WT, there was obvious turbidity in wells supplemented with 1,024 mg/L BBH, indicating a MIC of BBH larger than 1,024 mg/L, against this strain.

^d^ Original, the original stock of BCE prepared from the YPD culture of bacteria (see materials and methods).

**Table 4:** Phenotypes of the EBU45301 *tolC* mutant in drug susceptibility (disk/well-diffusion method).

Antimicrobial	Diameter of the inhibition zone (mm) for							
	WT	*tolC*	*tolC*/comp^a^	*tolC*/vect^b^	*p*-1^c^	*p*-2^c^	*p*-3^c^	*p*-4^c^
ß-lactam								
Piperacillin	28.5 ± 0.5	33.5 ± 1.0	28.3 ± 0.6	34.3 ± 1.6	0.0015	0.0015	0.0037	0.4883
Cefuroxime	22.8 ± 1.0	26.2 ± 1.5	22.2 ± 0.3	27.7 ± 1.3	0.0354	0.0112	0.0018	0.2595
Cefoperazone	30.7 ± 1.1	34.3 ± 1.2	29.2 ± 0.8	32.5 ± 0.5	0.0180	0.0030	0.0032	0.0651
Ceftriaxone	29.0 ± 2.0	28.8 ± 1.8			0.9189			
Aminoglycoside								
Gentamicin	19.5 ± 0.5	22.1 ± 1.2	19.6 ± 0.4	22.0 ± 0.5	0.0290	0.0270	0.0030	0.9126
Kanamycin	19.8 ± 0.3	22.8 ± 0.3	18.8 ± 0.8	21.7 ± 0.8	0.0002	0.0011	0.0105	0.0686
Amikacin	21.7 ± 0.6	24.3 ± 1.2	20.2 ± 0.3	23.8 ± 0.3	0.0232	0.0037	0.0001	0.5072
Streptomycin	16.2 ± 0.3	18.5 ± 0.5	17.0 ± 0.5	20.3 ± 0.6	0.0022	0.0213	0.0016	0.0142
Tetracycline								
Tetracycline	23.7 ± 0.6	27.5 ± 1.3	24.3 ± 0.6	27.5 ± 0.5	0.0100	0.0191	0.0020	1.0000
Minocycline	17.2 ± 0.8	24.3 ± 1.5	17.3 ± 0.3	22.8 ± 1.6	0.0019	0.0015	0.0043	0.3063
Doxycycline	16.3 ± 0.8	24.5 ± 1.0	18.3 ± 1.0	26.5 ± 0.5	0.0004	0.0018	0.0003	0.0363
Fluoroquinoline								
Ciprofloxacin^d^	26.2 ± 0.3	29.7 ± 1.2	25.7 ± 0.3	28.8 ± 0.8	0.0070	0.0043	0.0026	0.3560
Norfloxacin^d^	28.2 ± 0.6	31.2 ± 1.3	27.7 ± 0.8	29.7 ± 0.6	0.0199	0.0146	0.0224	0.1338
Levofloxacin	32.3 ± 0.8	37.2 ± 0.3	27.8 ± 2.8	36.8 ± 0.8	0.0005	0.0267	0.0055	0.5185
Phenicol								
Florfenicol	25.3 ± 1.5	35.5 ± 0.9	24.7 ± 1.3	35.3 ± 0.6	0.0006	0.0003	0.0002	0.7953
Folate pathway antagonist								
SXT^e^	25.0 ± 1.8	30.7 ± 0.8	24.7 ± 2.1	29.3 ± 1.9	0.0074	0.0094	0.0453	0.3211
Macrolide								
Erythromycin	ND^f^	17.8 ± 0.8	ND	17.7 ± 1.2	NA^f^	NA	NA	0.8450
Azithromycin	16.2 ± 0.8	19.7 ± 0.3	16.0 ± 0.4	20.0 ± 0.9	0.0018	0.0003	0.0020	0.5614
Lincosamide								
Lincomycin	None	Exist, ND	None	Exist, ND	NA	NA	NA	NA
Clindamycin	ND	18.0 ± 2.0	ND	16.7 ± 2.3	NA	NA	NA	0.4918
BCE	7.2 ± 0.2	10.4 ± 0.3	7.8 ± 0.1	11.0 ± 0.3	0.0001	0.0001	0.0001	0.0872

^a^ For tests with tetracycline, doxycycline, and BCE, the *tolC*/comp (complementation) strain used was *tolC*/pBYL030. For the rest cases, the *tolC*/complementation strain used was *tolC*/pBYL024.

^b^ For tests with tetracycline, doxycycline, and BCE, the *tolC*/vect (vector) strain used was *tolC*/pBYL031. For the rest cases, the *tolC*/vector strain used was *tolC*/pACYC184.

^c^ *p*-1, the *p*-value comparing *tolC* with WT; *p*-2, the *p*-value comparing *tolC*/complementation with *tolC*; *p*-3, the *p*-value comparing *tolC*/vector with *tolC*/complementation; *p*-4, the *p*-value comparing *tolC*/vector with *tolC*.

^d^ In the cases of ciprofloxacin or norfloxacin against the *tolC* ^+^ strains, an inhibition zone with complete none growth of bacteria (inner) and an inhibition zone with limited growth of bacteria (outer) were observed (**Figure S1**). Here, we considered both these areas as the inhibition zones for diameter determination.

^e^ SXT, trimethoprim-sulfamethoxazole.

^f^ ND, not determine-able: In the cases of erythromycin against the *tolC* ^+^ strains, the inhibition zones were not big enough for precise determination of diameters; In the cases of lincomycin against the *tolC* ^-^ strains and clindamycin against the *tolC* ^+^ strains, the inhibition zones were not regular enough for precise determination of diameters. NA, not applicable.

Berberine is known as a plant-isolated natural product exhibiting antibacterial activity against nosocomial pathogens like *Acinetobacter baumannii* and *Pseudomonas aeruginosa*. Also, berberine (hydrochloride) was considered as a substrate of the AcrB-homologous transporters in *A. baumannii* and *P. aeruginosa* (Morita et al., 2016; Li et al., 2021b). Here, we assessed the activity of BBH against the EBU45301 strains, and found that there is an at least 2-fold decrease in the MIC of BBH for *tolC*, compared with WT (**Table 3**). This result indicated that BBH displays intrinsic antibacterial activity for EBU45301 and can interact with one or more TolC-related efflux pumps in this bacterium.

It has been well characterized that *Bacillus* strains are able to synthesize various secondary metabolites, including fengycin, iturin, surfactin, difficidin, bacilysin, bacillibactin, bacillaene, amylocyclicin, subtilosin A, sublancin, and macrolactin, that possess antibacterial activity (Wilson et al., 1987; Scholz et al., 2014; Cavera et al., 2015; Liu et al., 2019; de Souza Freitas et al., 2020; Lv et al., 2020; Li et al., 2021a; Chakraborty et al., 2022; Erega et al., 2022; Zhou et al., 2022). There is a recent study showing that the transportation of bacilysin and bacillaene can be affected by RND-type transporters in *Campylobacter jejuni* (Erega et al., 2022). In the present study, we used the crude extract from strain GHZJ-1, which is a laboratory stock initially identified as *Bacillus* sp. and found exhibiting anti-yeast activity (data not shown), as one antimicrobial agent used for the susceptibility tests with the EBU45301 strains, see materials and methods for details. Obviously, comparing with the commercial antibiotic products, BCE used here was a crude agent and its exact concentration was difficult to determine. Thus, we first tested the MIC of BCE prepared from different batches with the EBU45301 strains. Our data revealed that BCE displays antibacterial activity against EBU45301 and that its MICs against WT and *tolC* are 0.031x original (BCE stock) and 0.016x original, respectively, no matter which batch of the BCE product was used (**Table 3**). Moreover, using the well-diffusion method, BCE formed larger inhibition zones for *tolC* than that for WT (10.4 mm vs. 7.2 mm; **Table 4**), and the variations among different batches were highly small (**Figure S2**), indicating that the antibacterial activity of BCE against EBU45301 is fairly stable. These results suggested the existence of the TolC pump-transported antibacterial metabolite(s) within BCE, like that observed in *C. jejuni* (Erega et al., 2022). Undoubtedly, identification of these specific metabolite(s) warrants more experiments.

The increased susceptibility to antimicrobial agents in the *tolC* mutant was complemented *via* introduction of the wild-type *tolC* (**Tables 3**, **4**, **Figures S1**, **Figure S2**), which confirmed the TolC effect on the susceptibility profile in this organism. Note that some of the complementation performed using plasmid pBYL024 is not exactly complete. This is likely due to the slight growth defect in strains harboring the pACYC184-based plasmids (**Figure S3**), which was not observed for strains carrying the plasmids (pBYL030-033) whose introduction was facilitated *via* integration into the chromosome (data not shown).

### Synergistic effects combining BCE and other TolC-affected antibiotics

**Figure 2:**
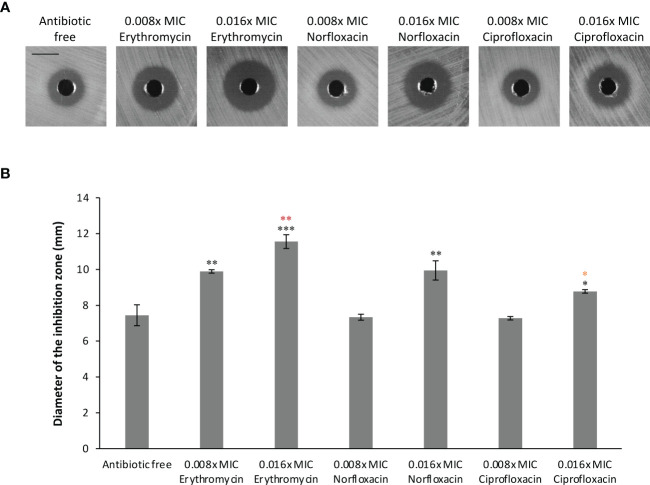
Synergistic effects combining BCE and erythromycin, norfloxacin, or ciprofloxacin against EBU45301. The susceptibility of WT to BCE was tested in the presence of different concentrations (fraction of MIC) of erythromycin, norfloxacin, or ciprofloxacin as indicated, using the well-diffusion method (see materials and methods). The inhibition zones formed by different BCE/antibiotic combinations against WT **(A)** were observed and determined for diameters **(B)**. The shown images are a representative of three independent experiments. All the black bars represent 10 mm. The diameter of inhibition zones was calculated as average ± SD of three independent experiments. * (black), *p* ≤ 0.05; ** (black), *p* ≤ 0.01; *** (black), *p* ≤ 0.001 comparing the inhibition-zone diameter with that for BCE against WT, in the absence of antibiotic. ** (red), *p* ≤ 0.01 comparing the inhibition-zone diameter for BCE in combination with 0.016x MIC erythromycin with that for BCE in combination with 0.008x MIC erythromycin. * (orange), *p* ≤ 0.05 comparing the inhibition-zone diameter for BCE in combination with 0.016x MIC ciprofloxacin with that for BCE in combination with 0.016x MIC norfloxacin.

The altered susceptibility to BCE observed in the *tolC* mutant suggests that there are substances capable of traveling through one or more TolC-mediated efflux pumps in this extract (**Tables 3**, **4**). Thus, BCE can be considered as a competitor of some other TolC-affected antibiotics for the capacity of efflux pumps regarding TolC. Besides, BCE is likely to contain lipopeptides, for instance, surfactin, that are able to change the cell-wall permeability and therefore potentiate the activity of other antibiotics against gram-negative bacteria (Liu et al., 2019). Taken together, we hypothesized that combinations of BCE and some TolC-affected antibiotics can confer synergistic effects against EBU45301. Here, we selected erythromycin, norfloxacin, and ciprofloxacin, whose MICs were decreased the most in EBU45301 strains devoid of *tolC* (**Table 3**), as the agents respectively used in association with BCE, for synergistic assays. As shown in **Table 5**, weak synergistic effects were observed for combinations of BCE/erythromycin: in the presence of 0.5x MIC BCE, the MIC of erythromycin against EBU45301 WT was 384 mg/L (0.25x MIC), and *vice versa*. However, such synergistic effects were not observed for combinations of BCE/norfloxacin or ciprofloxacin (**Table 5**). It has been characterized that the checkerboard method is unable to fully test synergistic effects due to its twofold dilution feature (Grimsey et al., 2020). Thus, we also performed synergistic assays using the well-diffusion method, as previously described (Grimsey et al., 2020). Our results showed that in the presence of 12 mg/L (0.008x MIC) erythromycin, the antibacterial activity of BCE against WT is increased, compared with that in the absence of antibiotics (9.9 mm vs. 7.4 mm; **Figure 2**). This increased phenotype in BCE activity was enhanced in the presence of 24 mg/L (0.016x MIC) erythromycin (11.6 mm vs. 7.4; **Figure 2**). Similarly, the BCE activity against WT was elevated in the presence of 0.006 mg/L (0.016x MIC) norfloxacin (9.9 mm vs. 7.4 mm) or 0.5 µg/L (0.016x MIC) ciprofloxacin (8.8 mm vs. 7.4 mm), and yet the existence of neither of these antibiotics at 0.008x MIC (0.003 mg/L or 0.25 µg/L) could potentiate the BCE activity (**Figure 2**). It appeared that 0.016x MIC norfloxacin increases the BCE activity slightly more than 0.016x MIC ciprofloxacin (**Figure 2**). These results revealed that at sub-MICs, erythromycin, norfloxacin, and ciprofloxacin can all enhance the BCE activity inhibiting EBU45301 with erythromycin being the most efficient. Our findings also supported that BCE used in this study can serve as a resource developing novel antibacterial agents or antibiotic adjuvants against *E. bugandensis*. Of course, analysis and isolation of the active substances within BCE is inevitable in future studies.

**Table 5:** Synergistic tests using BCE in combination with certain TolC-affected antibiotics (checkerboard method).

Antibiotic	Concentration of BCE (Fraction of MIC^a^)	MIC (mg/L) against WT
Erythromycin	None	1,536
	0.125x	1,536
	0.25x	768
	0.5x	384
Norfloxacin	None	0.384
	0.125x	0.384
	0.25x	0.384
	0.5x	0.384
Ciprofloxacin	None	0.032
	0.125x	0.032
	0.25x	0.032
	0.5x	0.032

^a^ Note that the MIC of BCE against WT was 0.031x original (see **Table 3**).

## Discussion

*E. bugandensis* is an emerging pathogen in which MDR strains are frequently isolated, which poses serious problems in their treatments during nosocomial infections (Doijad et al., 2016; Pati et al., 2018; Singh et al., 2018; Falgenhauer et al., 2019; Matteoli et al., 2020; Moon et al., 2021). Genomic analyses have demonstrated that many efflux pump genes are included in genomes of *E. bugandensis* strains (Matteoli et al., 2020), and yet a comprehensive description of the relationship between efflux pumps and antibiotic susceptibility/resistance in this species has not been reported. Here, we studied the effect of TolC, an outer-membrane component of different efflux pumps, on the susceptibility to a range of 26 antimicrobial agents in a newly reclassified *E. bugandensis* strain, CMCC(B) 45301 (EBU45301). We also investigated whether particular combinations of the TolC-affected natural product/antibiotic exhibited synergistic effects. The goal of this study is to elucidate the physiological role of TolC in the antimicrobial susceptibility profile of EBU45301, which might contribute to the development and application of new therapeutic options against (MDR) *E. bugandensis*.

In the present study, we sequenced the complete genome and plasmid harbored in the *Enterobacter* strain CMCC(B) 45301 (GenBank accession No. CP097254-CP097255), and reidentified this organism from *E. cloacae* to *E. bugandensis*, on the basis of the ANI of its genomic assembly against the best-matching type-strain assembly of *E. bugandensis* (Ciufo et al., 2018). This provides a full-detail reference of the EBU45301 genome, which is still scarce within species *bugandensis*. Using this reference sequence, we identified the specific EBU45301 homologues of the respective *tolC* dependent efflux-pump genes characterized in *E. coli* and *E. cloacae* (**Table 1**). In addition, some putative efflux-pump genes whose products display homology with components involved in the known TolC-related efflux pumps were located in the EBU45301 genome (**Tables S1**-**3**). The specific homologues for some of these genes were not found in the genome of *E. cloacae* ATCC 13047 (**Tables S2**, **Table S3**), indicating that they are not part of the core resistome in genus *Enterobacter*. All these comparative results suggested a significant role of TolC in EBU45301. Intriguingly, we noticed that genes of M1V99_14575, M1V99_14580 (**Table S2**), M1V99_14525, M1V99_14530, and M1V99_14535 (**Table S3**) are included within a ~90,000 bp sequence (Position 2864027-2954705) in the EBU45301 genome that is not highly identical to any sequences but one within the genome of *E. ludwigii* strain UW5 (Position 3536652-3627328), BLASTing it against the NCBI database. Moreover, several hypothetical MF transporters are encoded in this big-chunk DNA fragment, suggesting a unique armory of MDR mechanisms in EBU45301. More research regarding this fragment may provide insights into areas of how MDR genes are transferred horizontally among environmental strains.

We constructed a deletion mutant for *tolC* and subjected it to antimicrobial susceptibility tests, along with the wild type. It is found that, following the guidelines of breakpoints recommended for *Enterobacteriaceae*, the wild-type EBU45301 is resistant to cefazolin, weakly susceptible to streptomycin, doxycycline, minocycline, and chloramphenicol, susceptible to piperacillin, cefuroxime, ceftazidime, ceftriaxone, cefoperazone, imipenem, amikacin, gentamicin, kanamycin, tetracycline, norfloxacin, ciprofloxacin, levofloxacin, and trimethoprim-sulfamethoxazole (**Tables 2**-**4**). Furthermore, the *tolC* deletion resulted in the increased susceptibility to all the assayed antimicrobial agents, except cefazolin, ceftazidime, ceftriaxone, and imipenem, in EBU45301 (**Tables 2**-**4**). However, it has been described that efflux pumps regarding TolC are likely to be capable of transporting cefazolin, ceftazidime, ceftriaxone, and imipenem in *E. coli* or certain *Enterobacter* species (Chang et al., 2007; Perez et al., 2007; Moosavian et al., 2021). The phenotype unresponsive to the *tolC* deletion for these four ß-lactams in EBU45301 indicated that the deletion phenotype is not obvious enough, as the overexpression phenotype, or that even similar efflux pumps identified in closely-related species vary in their specific substrates (Perez et al., 2007; Chang et al., 2007; Guerin et al., 2016). Despite of this, the effect of TolC-involved efflux pumps on other tested antibiotics belonging to ß-lactam, aminoglycoside, tetracycline, fluoroquinolone, phenicol, macrolide, lincosamide, and folate pathway antagonist observed in EBU45301 is similar to that found in *E. coli* or *Enterobacter*, as listed in **Table S4**. Moreover, the *tolC* strain exhibited increased susceptibility to the natural product-based agents, BBH and BCE (**Tables 3**, **4**), in EBU45301, which to our best knowledge has not been characterized for *Enterobacter*.

Berberine or BBH can be used as an efflux-pump inhibitor in association with some efflux-pumped antibiotics against MDR *A. baumannii* and *P. aeruginosa* (Morita et al., 2016; Li et al., 2021b). However, there was no synergistic effects detected combining BBH and the respective TolC-affected antibiotics, erythromycin, norfloxacin, ciprofloxacin, chloramphenicol, and tetracycline, in EBU45301 (data not shown). This might be due to the just-constitutive expression of the efflux pump genes in EBU45301, different from the strains previously used for testing the activity of certain combinations of efflux-pumped antibiotic/efflux-pump inhibitor, which are either spontaneously or artificially MDR (Morita et al., 2016; Grimsey et al., 2020; Li et al., 2021b). Therefore, in our future studies, synergistic tests using BBH and other TolC-affected antibiotics will be performed with artificial MDR strains, such as the *ramR* mutant, in EBU45301. Given the fact that BCE includes substances TolC efflux pump-transported, we also assayed the synergistic effects using BCE in combination with erythromycin, norfloxacin, or ciprofloxacin, three antibiotics the susceptibility to which was affected the most in *tolC*. Our results demonstrated that erythromycin, norfloxacin, and ciprofloxacin can potentiate the activity of BCE against EBU45301, at their sub-MICs (**Table 5** and **Figure 2**). Erythromycin exhibited the highest ability to potentiate the BCE activity against EBU45301, and norfloxacin seemed to be more efficient as an adjuvant for BCE than ciprofloxacin (**Figure 2**). It appeared that the more an antibiotic is affected by TolC, the better a synergistic compound for BCE against EBU45301 it is. To elucidate this, more experiments are required. In terms of BCE, we surely can consider it as a reservoir isolating for active substances as the antibacterial agents or antibiotic adjuvants against *E. bugandensis* in the future. However, we still have a long way to go. First, whole genome sequencing of the *Bacillus* strain used in this study is warranted for typing it to a specific species and providing a genetic reference including clusters encoding different secondary metabolites. Secondly, antibacterial substances, especially those with the efflux-pumped features, like bacilysin and bacillaene in *C. jejuni* (Erega et al., 2022), and the antibiotic-adjuvant features, like surfactin in *E. coli* and subtilosin A in *Gardnerella vaginalis* (Cavera et al., 2015; Liu et al., 2019), are required to be identified and isolated for more specific studies. Finally, experiments about genetic modification or incubation conditions promoting the production of useful metabolites of our *Bacillus* strain are desired, for a more efficient application of this organism.

In summary, the present study revealed that TolC is involved in the efflux of a broad range of antimicrobial agents encompassing antibiotic families of ß-lactam, aminoglycoside, tetracycline, fluoroquinolone, phenicol, macrolide, lincosamide, and folate pathway antagonist, and natural product-based agents, like BBH and BCE, in EBU45301. In addition, certain combinations of the TolC-affected agents, namely BCE and erythromycin, norfloxacin, or ciprofloxacin, displayed synergistic effects combating EBU45301. This investigation provided a comprehensive description of the TolC effect on multidrug susceptibility/resistance in *E. bugandensis*, which can be used as a reference for the future emergence of MDR isolates belonging to this species and as research endorsing the development of more therapeutic strategies against this opportunistic pathogen.

## Materials and methods

### Bacterial strains and growth conditions

All the bacterial strains used in the present study are listed in **Table 6**. Except where indicated, the *E. coli* and EBU45301 strains were grown in LB broth or agar (Solarbio, Beijing, China) at 37°C aerobically. If required, 12.5 mg/L chloramphenicol (Solarbio) or 10 mg/L tetracycline (Solarbio), was used for the *E. coli* and EBU45301 strains.

**Table 6:** Bacterial strains used in this study.

Strain/Plasmid	Genotypes^a^	Source
Strain		
EBU45301 (WT)	The *E. bugandensis* CMCC(B) 45301 strain	CMCC
S17-1 λpir/pBYL009	The *E. coli* S17-1 λpir strain transformed with pBYL009 (Cm^r^)	This study
*tolC*	The clean deletion mutant for *tolC* in EBU45301 (mediated by pBYL009)	This study
DH5α/pBYL024	The *E. coli* DH5α strain transformed with pBYL024 (Cm^r^)	This study
*tolC*/pBYL024	The *tolC* strain transformed with pBYL024 (Cm^r^)	This study
*tolC*/pACYC184	The *tolC* strain transformed with pACYC184 (Cm^r^, Tc^r^)	This study
S17-1 λpir/pBYL030	The *E. coli* S17-1 λpir strain transformed with pBYL030 (Cm^r^)	This study
S17-1 λpir/pBYL031	The *E. coli* S17-1 λpir strain transformed with pBYL031 (Cm^r^)	This study
*tolC*/pBYL030	The *tolC* strain with pBYL030 integrated into the genome (Cm^r^)	This study
*tolC*/pBYL031	The *tolC* strain with pBYL031 integrated into the genome (Cm^r^)	This study
S17-1 λpir/pBYL032	The *E. coli* S17-1 λpir strain transformed with pBYL032 (Tc^r^)	This study
S17-1 λpir/pBYL033	The *E. coli* S17-1 λpir strain transformed with pBYL033 (Tc^r^)	This study
*tolC*/pBYL032	The *tolC* strain with pBYL032 integrated into the genome (Tc^r^)	This study
*tolC*/pBYL033	The *tolC* strain with pBYL033 integrated into the genome (Tc^r^)	This study
*Bacillus* sp. GHZJ-1	The *Bacillus* strain GHZJ-1 isolated from a waste-water pond of Panjin Guanghe Crab Industry Co., Ltd., Panjin, China	Lab stock
Plasmid		
pRE112	Cloning vector (Cm^r^)	Edwards et al., 1998
pACYC184	Cloning vector (Cm^r^, Tc^r^)	Chang and Cohen, 1978
pDMS197	Cloning vector (Tc^r^)	Edwards et al., 1998
pBYL009	The Δ*tolC* fragment cloned in pRE112 (Cm^r^)	This study
pBYL024	The *tolC* fragment cloned in pACYC184 (Cm^r^)	This study
pBYL030	The *tolC* fragment cloned in pRE112 (Cm^r^)	This study
pBYL031	The *tolC* ^UP^ fragment cloned in pRE112 (Cm^r^)	This study
pBYL032	The *tolC* fragment cloned in pDMS197 (Tc^r^)	This study
pBYL033	The *tolC* ^UP^ fragment cloned in pDMS197 (Tc^r^)	This study

^a^ Cm^r^, chloramphenicol resistant; Tc^r^, tetracycline resistant.

### Whole genome sequencing of EBU45301

In this study, the whole genome sequencing of EBU45301 was accomplished using a combination of the Pacbio and Illumina platforms. For sequencing performed by the Pacbio platform, the total DNA isolated from EBU45301 was subjected to construction of the 10Kb (kilo base) SMRTbell library, using the SMRTbell™ Template kit version 1.0. The constructed library was tested using Qubit and Agilent 2100 for its quality and fragment-size, respectively, followed by the sequencing conducted *via* PacBio Sequel. For sequencing performed by the Illumina platform, the total DNA was randomly broken into fragments of ~350 bp using the Covaris shearing instrument, and a library of these fragments was constructed according to the manufacturer’s instructions of the NEBNext^®^Ultra™ DNA Library Prep Kit for Illumina (NEB, MA, USA). Similarly, the Illumina library was subjected to quality tests by Qubit and fragment-size checking by Agilent 2100, which was followed by the sequencing conducted *via* Illumina NovaSeq PE150. The original sequencing data were filtered for the elimination of low-quality reads and adaptors, and the obtained clean data were assembled using the software SMRT Link version 5.0.1, as previously described (Ardui et al., 2018; Reiner et al., 2018).

### Construction of phylogenic tree based on the *tolC* gene sequences

The *tolC* gene sequences were retrieved from the genomic sequences of the type or recommended reference strains representing their species in *Enterobacter*, as indicated in **Figure 1A**. These *tolC* sequences were aligned by MUSCLE (Codons) in MEGA version 11, the result of which was subsequently saved and used for phylogenic-tree construction *via* the method of maximum-likelihood. The bootstrap values were determined based on 500 replications.

### Alignment of the amino acid sequences of TolC from different *Enterobacter* strains

The alignment of the nucleotide sequences of *tolC* described above (for the phylogenic tree) was translated into amino acid sequences using the translating function for alignments in MEGA version 11. The resulted alignment of the amino acid sequences was saved as a FASTA file. This file was opened, edited for display settings, and finally exported as a figure using the software DNAMAN version 10.

### Construction of strains for the *tolC* deletion and complementation

To construct a clean deletion for *tolC* in EBU45301, a pRE112-based system was used as previously described (Edwards et al., 1998). Generally, the upstream and downstream of the EBU45301 *tolC* gene were PCR amplified using the primer pairs *tolC* ^UP^-F: 5’-GAG CTC CCA GAT AGC TCA ACA CCG GT-3’/*tolC* ^UP^-R: 5’-GGC GTG ATA ACA CTC TTG CAT TCC TTG TTG TGA AG-3’ and *tolC* ^DOWN^-F: 5’-CAA CAA GGA ATG CAA GAG TGT TAT CAC GCC CTC TC-3’/*tolC* ^DOWN^-R: 5’-GGT ACC CAT CAT GTA ACC TGC CAT TAA T-3’, respectively. These two DNA fragments were subsequently spliced by overlap extension (SOE) PCR (Thornton, 2016), to obtain the DNA product of Δ*tolC*, flanked by the built-in sequences of Sac I and Kpn I. The Δ*tolC* fragment was subcloned into pRE112 between Sac I and Kpn I, resulting in plasmid pBYL009. pBYL009 was then transformed into *E. coli* S17-1 λpir, and the resultant strain was used along with the wild-type EBU45301 for conjugation performed at 30°C. The conjugants of EBU45301 were selected as chloramphenicol-resistant (Cm^r^) and cefazolin-resistant (Cz^r^) colonies in LB plates supplemented with 20 mg/L cefazolin (Apexbio, TX, USA) and 12.5 mg/L chloramphenicol. These conjugants were subsequently streaked on LB plates supplemented with 6% (w/v) sucrose (Solarbio) for separated single colonies, randomly picking and patching of which were conducted simultaneously on LB plates supplemented with or without chloramphenicol. The sucrose-resistant but chloramphenicol-sensitive colonies were further subjected to PCR tests using the primer pair *tolC* ^UP^-F/*tolC* ^DOWN^-R, and isolates of the *tolC* mutant were screened out as the ones from which a ~1100 bp product was amplified by this primer pair.

To complement the *tolC* deletion, fragment of the wild-type *tolC* gene was PCR amplified using the primer pair *tolC*-F1: 5’-CCC GTC CTG TGG ATC CTC GCC CTC TTC GAT CAT CC-3’/*tolC*-R1: 5’-CCG GCG TAG AGG ATC CAT CAT GTA ACC TGC CAT TAA T-3’. In this way, the *tolC* fragment was flanked by the sequences (underlined) homologous with those in plasmid pACYC184. This *tolC* fragment and the linear pACYC184, digested at site BamH I, were ligated according to the manufacturer’s instructions of the ClonExpress Ultra One Step Cloning Kit (Vazyme Biotech Co., Ltd, Nanjing, China), and the resulted plasmid was designated as pBYL024. The *tolC* mutant was subsequently transformed with pBYL024, and the *tolC*/pBYL024 strains were isolated as Cm^r^ colonies. In the meanwhile, the *tolC* mutant was transformed with pACYC184, and the Cm^r^ transformants were isolated and used as a control strain for the empty vector. Given that the tetracycline-resistant (Tc^r^) feature of pACYC184 is not suitable for the susceptibility tests using antibiotics like tetracycline and doxycycline, the fragment including gene *tolC* and its upstream and downstream was inserted in pRE112, to obtain an alternative complementing plasmid. Briefly, the *tolC* fragment was PCR amplified using the primer pair *tolC*-F2: 5’-TAT CGC ATG CGG TAC CTC GCC CTC TTC GAT CAT CC-3’/*tolC*-R2: 5’-TTC TTC TAG AGG TAC CAT CAT GTA ACC TGC CAT TAA T-3’. The sequences homologous with those in pRE112 have been underlined. This fragment was then subcloned in pRE112 at site Kpn I, in the same manner as that described for pBYL024, and the resulted plasmid was designated as pBYL030. As shown in **Figure S4**, the suicide plasmid pBYL030 is introduced and integrated into the genome of the *tolC* mutant, *via* the homologous recombination during the conjugation of S17-1 λpir/pBYL030 and *tolC*. The *tolC*/pBYL030 strain used in this study was selected as Cm^r^ and Cz^r^ colonies in which the homologous recombination was occurred within the upstream of *tolC* (**Figure S4**). To check this, PCR tests using the primer pair *tolC*-CF:5’-AGG GCG GTC AGG TAA ACT CT-3’/*tolC*-CR1: 5’-GCG TGT TAC GGT GAA AAC CT-3’ were performed, as described in **Figure S4**. The vector-control strain, *tolC*/pBYL031, was constructed in the same manner as that described for *tolC*/pBYL030, except the different primer pair used for cloning of the upstream of *tolC* (*tolC* ^UP^; **Figure S5**). This primer pair was *tolC*-F2/*tolC*-R3: 5’-TTC TTC TAG AGG TAC TTG CAT TCC TTG TTG TGA AG-3’. In addition, to perform complementation suitable for the susceptibility tests using chloramphenicol, the other alternative complementing plasmid, pBYL032, and its vector-control, pBYL033, were obtained, in the same manner as that described for pBYL030 and pBYL031, except that pDMS197 was used instead of pRE112 for cloning. Strain *tolC*/pBYL032 and *tolC*/pBYL033 were selected as Tc^r^ and Cz^r^ colonies, *via* the same pipelines constructing *tolC*/pBYL030 and *tolC*/pBYL031 (**Figures S4**, **S5**). To check the desirable *tolC*/pBYL032 isolates, the primer pair used for PCR was *tolC*-CF/*tolC*-CR2: 5’-GAC AGC ATC GCC AGT CAC TA-3’.

### Initial identification of the *Bacillus* sp. GHZJ-1 strain

The bacterial strain GHZJ-1 was isolated from a waste-water pond possessed by the Panjin Guanghe Crab Industry Co., Ltd and exhibited anti-yeast activity in other studies of our group (data not shown). To identify this bacterium, the partial region of its 16S rDNA gene was PCR amplified using the universal primer pair 27F/1492R. The PCR products were subjected to sanger sequencing, and the 16S rDNA sequence of GHZJ-1 was subsequently assembled (GenBank accession No. OP316901). BLASTing this sequence against the NCBI database, its homologues in strains identified as *Bacillus amyloliquefaciens* and *Bacillus velezensis* were shown with the highest score (bits), 100% identities and 100% query cover. Thus, GHZJ-1 was initially identified as a *Bacillus* strain.

### Preparation of BCE

To prepare the crude extract of *Bacillus* sp. GHZJ-1, the strain was grown in YPD broth (Solarbio) at 28°C with aeration for 72 hours (h). Subsequently, the YPD culture of GHZJ-1 was subjected to 1.5-h sonication (every 15-second sonication process was followed by a 15-second pause) and centrifugation at 4°C, to obtain the supernatant. This supernatant was further sterilized *via* the 0.22-micrometer filters (Sangon Biotech, Shanghai, China) and finally used as the BCE agent (original) in susceptibility tests against EBU45301. In this manner, three independent batches of BCE were prepared for the susceptibility tests. The BCE stocks were preserved at -80°C and thawed on ice before use.

### Antimicrobial susceptibility test

In this study, the MICs of piperacillin, cefazolin, ceftazidime, imipenem, gentamicin, kanamycin, tetracycline, norfloxacin, ciprofloxacin, chloramphenicol, and erythromycin were determined using the method of broth microdilution, according to the standards for the dilution methods provided by CLSI, 2018a. Briefly, the tested antibiotics at desired concentrations were prepared using cation-adjusted Mueller-Hinton broth (CAMHB; Solarbio) in a twofold-dilution manner and aliquoted in 96-well microtiter plates. The final cell density of the inoculum for each well was 5x 10^5^ CFU/milliliter (mL). The microtiter plates were incubated at 37°C for 18 h in an ambient air incubator, and the MIC of antibiotics was measured as their lowest concentration that completely inhibit bacteria growth (CLSI, 2018a). The concentrations used for the tested antibiotics ranged as follows: piperacillin (Solarbio), 64-0.031 mg/L; cefazolin (Apexbio), 512-0.5 mg/L; ceftazidime (Solarbio), 64-0.031 mg/L; imipenem (Solarbio), 16-0.008 mg/L; gentamicin (Solarbio), 32-0.016 mg/L; kanamycin (Solarbio), 32-0.016 mg/L; tetracycline (Solarbio), 32-0.016 mg/L; norfloxacin (Solarbio), 6.144-0.0015 mg/L; ciprofloxacin (Solarbio), 0.512-0.00025 mg/L; chloramphenicol (Solarbio), 128-0.06 mg/L; erythromycin (Solarbio), 6,144-0.75 mg/L. Three independent experiments were performed to determine the MICs of these antibiotics, and in every independent experiment, the tests for each concentration used were conducted in triplicates. The disk-diffusion tests were performed according to the standards for the methods of disk diffusion provided by CLSI, 2018b, using the antibiotic disks (BKMAM, Hunan, China) as follows: piperacillin, 100 micrograms (µg); cefuroxime, 30 µg; ceftriaxone, 30 µg; cefoperazone, 75 µg; amikacin, 30 µg; gentamicin, 10 µg; kanamycin, 30 µg; streptomycin, 10 µg; tetracycline, 30 µg; minocycline, 30 µg; doxycycline, 30 µg; levofloxacin, 5 µg; florfenicol, 30 µg; trimethoprim-sulfamethoxazole, 1.25-23.75 µg; erythromycin, 15 µg; azithromycin, 15 µg; lincomycin, 2 µg; clindamycin, 2 µg. Briefly, the tested bacteria were seeded in the Mueller-Hinton agar (MHA; Solarbio) plates *via* evenly coating the plates with streaks of the inoculum (0.5 McFarland standard). The antibiotic disks were then placed properly in the inoculated MHA plates, which was followed by 18-h incubation at 37°C of these plates in an ambient air incubator (CLSI, 2018b). The well-diffusion tests were performed for ciprofloxacin and norfloxacin. Basically, the MHA plates were evenly coated with bacterial cultures, as that described for the disk-diffusion tests (CLSI, 2018b). Afterwards, cylinder wells with 6 mm-diameter base sides were drilled in the inoculated agar with a height of approximately 3 mm. 75 microliters (µL) of 0.16 mg/L ciprofloxacin or 1.92 mg/L norfloxacin (both diluted in CAMHB) was aliquoted into each well for the tests. Finally, all the treated agar plates were incubated at 37°C for 18 hours in an ambient air incubator, to observe the inhibition zones. 75 µL CAMHB was used as the negative control for each well. Diameter of the inhibition zone for well-diffusion tests was determined as the value subtracting the well diameter (6 mm) from the whole diameter for the inhibition circle. For each tested antibiotic in disk-diffusion and well-diffusion assays, diameter of the inhibition zone was measured as average ± SD based on three independent experiments. The comparative analyses of data were performed using student’s *t* tests. The susceptibility/resistance of EBU45301 to piperacillin, cefazolin, ceftazidime, imipenem, gentamicin, kanamycin, tetracycline, norfloxacin, ciprofloxacin, and chloramphenicol was determined according to the MIC-based breakpoints provided by CLSI, 2022. The susceptibility/resistance of EBU45301 to cefuroxime, ceftriaxone, cefoperazone, amikacin, streptomycin, minocycline, doxycycline, levofloxacin, and trimethoprim-sulfamethoxazole was determined according to the breakpoints based on the inhibition-zone diameters, provided by CLSI, 2022. For the susceptibility tests the readings of which were used for susceptibility/resistance determination, the *E. coli* ATCC 25922 strain was used as a quality-control strain (CLSI, 2022).

To assess the susceptibility to BBH in the EBU45301 strains, 18 mg/mL BBH was prepared using DMSO as the solvent. The MICs of BBH against the tested strains were determined using the broth-microdilution method as described above. In the meanwhile, the antibacterial activity of DMSO at the concentrations same as that contained in the serially diluted BBH was tested independently, to rule out the interference of DMSO in inhibition of EBU45301 growth. The concentrations of BBH used for the tests ranged from 2,048 mg/L to 4 mg/L.

The susceptibility of the EBU45301 strains to BCE was tested using both the broth-microdilution and well-diffusion method, as described above. To determine the MIC of BCE against the EBU45301 strains, the concentrations ranged from 0.5x original (BCE) to 0.001x original (BCE) were prepared by performing serial twofold dilutions with CAMHB and used for the tests. In the meanwhile, the antibacterial activity of YPD broth at the concentrations same as that contained in the serially diluted BCE was tested independently, to rule out the interference of YPD broth in inhibition of EBU45301 growth. Using the same batch of BCE (three batches in total), two independent experiments were performed for each of the EBU45301 strain, and in every independent experiment, the tests for each concentration used were conducted in triplicates. In the well-diffusion assays, 75 µL BCE (original) was aliquoted into each well, and 75 µL YPD broth was used as the negative control for each well. Three independent experiments were performed with each one using BCE from different batches for comparisons. To determine the diameter of the inhibition zones formed by BCE, the average reading for the three BCE batches used in the same independent experiment was calculated first, and the final value of diameter was determined as average ± SD based on the average readings for the three independent experiments. The comparative analyses of data were performed using student’s t tests.

### Synergistic assay

The synergistic assays testing BCE in association with erythromycin, norfloxacin, or ciprofloxacin against the EBU45301 WT were performed using both the checkerboard method and the well-diffusion method. The checkerboard arrangements for the tested combinations were in the same manner as previously described (MacNair et al., 2018). In the checkerboard assays, the fractional inhibitory concentrations of BCE, erythromycin, norfloxacin, and ciprofloxacin against WT were determined using the broth-microdilution protocols for these four antibiotics, respectively, as described above. To better assay the synergistic effects combining BCE and erythromycin, norfloxacin, or ciprofloxacin against WT, the well-diffusion protocol described for testing the antibacterial activity of BCE was used. The inhibition zones formed by BCE in the seeded MHA plates supplemented with 12 mg/L erythromycin, 24 mg/L erythromycin, 0.003 mg/L norfloxacin, 0.006 mg/L norfloxacin, 0.25 µg/L ciprofloxacin, 0.5 µg/L ciprofloxacin, or none antibiotics were observed and determined for diameters, as described above. The comparative analyses of data were performed using student’s *t* tests.

## Data availability statement

The datasets presented in this study can be found in online repositories. The names of the repository/repositories and accession number(s) can be found in the article/**Supplementary Material**.

## Author contributions

BL and JZ conducted the experiments. BL, JZ, and XL analyzed the data. BL and XL drafted the manuscript on the basis of the analyzed data. BL and XL designed the whole project. All authors contributed to the article and approved the submitted version.
