# Supplementary material for: A comprehensive description of the TolC effect on the antimicrobial susceptibility profile in Enterobacter bugandensis
Source: Front Cell Infect Microbiol. 2022 Dec 9;12:1036933. doi: 10.3389/fcimb.2022.1036933 (PMC9780596; doi:10.3389/fcimb.2022.1036933)
Supplement: Supplementary file 1 [file DataSheet_1.pdf]

Table S1. List of the EBU45301 genes sharing identities with some other RND efflux-pump sequences characterized in *Enterobacter cloacae* ECL13047[a]

Locus Tag EBU45301	Locus Tag ECL13047	Symbol	Description of gene product	Identities/positives (%)[b]
M1V99_00845	ECL_RS00280/ECL_00055	*eefA*	Periplasmic adaptor subunit of RND transporter	97/99
M1V99_00840	ECL_RS00275/ECL_00054	*eefB*	Permease subunit of RND transporter	98/99
M1V99_00835	ECL_RS00270/ECL_00053	*eefC*	Outer membrane subunit of efflux pump	95/98
M1V99_06175	ECL_RS15520/ECL_03150		Periplasmic adaptor subunit of RND transporter	89/93
M1V99_06180	ECL_RS15515/ECL_03149		Permease subunit of RND transporter	96/98
M1V99_10315	ECL_RS11005/ECL_02243		Periplasmic adaptor subunit of RND transporter	82/89
M1V99_10310	ECL_RS11010/ECL_02244		Permease subunit of RND transporter	88/93
M1V99_10495	ECL_RS10450/ECL_02124	*oqxA*	Periplasmic adaptor subunit of RND transporter	91/95
M1V99_10500	ECL_RS10455/ECL_02125	*oqxB*	Permease subunit of RND transporter	98/99
M1V99_12500	ECL_RS08665/ECL_01758		Permease subunit of RND transporter	88/94

[a] See reference 1.

[b] This result reveals the similarity of an EBU45301 amino acid sequence against its respective homologue in ECL13047. The query covers (%) of sequences used in alignments for each of the EBU45301 protein are all larger or equal to 99.

Table S2. List of the RND efflux-pump genes in EBU45301 that possess significant similarity with none of the *E. cloacae* ECL13047 genome

Locus tag	Description of gene product	Identities/positivies (%) with the EBU45301 protein[a]				
		AcrB	AcrD	AcrF	MdtB	MdtC
M1V99_14575	Permease subunit of RND transporter	26/45	28/47	26/46	29/49	29/49
M1V99_14705	Permease subunit of RND transporter	22/42	23/44	23/43	25/42	24/43
		AcrA	AcrE	MdtA		
M1V99_10805	Periplasmic adaptor subunit of RND transporter	24/40	33/46	25/41		

M1V99_14580	Periplasmic adaptor subunit of RND transporter	28/43	25/42	24/45
M1V99_14700	Periplasmic adaptor subunit of RND transporter	24/40	23/40	25/41

[a] The query covers (%) of sequences used in alignments for each of the protein of interest are all larger than 75, except for that comparing M1V99_10805 with AcrE (35) and M1V99_14580 with AcrA (47).

Table S3. List of the MF efflux-pump genes in EBU45301 that possess similarity with the respective EmrKY/EmrAB-TolC efflux-pump genes

Locus tag EBU45301	Locus tag ECL13047	Description of gene product	Identities/positivies (%)[a]	
			EmrK *Escherichia coli*	EmrA EBU45301
M1V99_07425	ECL_RS14265	HlyD family secretion protein	30/52	36/55
M1V99_10955	ECL_RS10265	HlyD family secretion protein	32/49	34/49
M1V99_14530	ND[b]	HlyD family efflux transporter periplasmic adaptor subunit	41/58	43/58
M1V99_01260	ECL_RS00830	HlyD family secretion protein	21/45	24/43
M1V99_03460	ECL_RS03535	HlyD family secretion protein	25/43	28/43
M1V99_09990	ECL_RS11385	HlyD family secretion protein	27/44	27/44
			EmrY *E. coli*	EmrB EBU45301
M1V99_07430	ECL_RS14260	Permease subunit of MF transporter	35/58	37/57
M1V99_10960	ECL_RS10260	MFS transporter	22/39	28/45
M1V99_14535	ND	Permease subunit of MF transporter	38/58	41/60
			TolC *E. coli*	TolC EBU45301
M1V99_07435	ECL_RS14255	Outer membrane subunit of efflux pump	23/39	23/41
M1V99_10950	ECL_RS10270	Outer membrane subunit of efflux pump	27/47	23/40
M1V99_14525	ND	Outer membrane subunit of efflux pump	25/44	23/45

[a] This result reveals the similarity of an EBU45301 amino acid sequence against its respective homologues as indicated. The query covers (%) of sequences used in alignments for each of the protein of interest are all larger than 75, except for that comparing M1V99_01260 with EmrK *E. coli* (61) or EmrA EBU45301 (61), M1V99_03460 with EmrA EBU45301 (73), M1V99_10960 with

EmrB EBU45301 (23), M1V99_10950 with TolC *E. coli* (32), and M1V99_14525 with TolC *E. coli* (41) or TolC EBU45301 (40).
[b] ND, non-detectable.

Table S4. List of antibiotics the susceptibility to which is affected by the TolC-involved efflux pumps in *E. coli* and *Enterobacter* strains

Bacterial species	Particular transporters responsible for the changed susceptibility to antibiotics		
E. coli	AcrAB (2-8)<br>Nitrocefin, cephalothin, cefamandole, cephaloridine, cefazolin, dicloxacillin, cloxacillin, oxacillin, azlocillin, mezlocillin, piperacillin, penicillin, ampicillin, erythromycin, ciprofloxacin, nalidixic acid, norfloxacin, trimethoprim, chloramphenicol, colistin, mitomycin C, fusidic acid, novobiocin, daunomycin, methotrexate, puromycin, doxorubicin	AcrAD (9-13)<br>Oxacillin, cloxacillin, nafcillin, carbenicillin, sulbenicillin, aztreonam, amikacin, gentamicin, tobramycin, kanamycin, neomycin, tetracycline, erythromycin, nalidixic acid, norfloxacin, novobiocin, puromycin, fosfomycin	AcrEF (9, 14)<br>Tetracycline, minocycline, doxycycline, erythromycin, nalidixic acid, norfloxacin, enoxacin, chloramphenicol, novobiocin, doxorubicin
	MdtEF (13, 15, 16)<br>Oxacillin, cloxacillin, nafcillin, erythromycin, ciprofloxacin, novobiocin, doxorubicin<br>MacAB (19)<br>Erythromycin, Clarithromycin, Oleandomycin	MdtABC (11, 17)<br>nalidixic acid, norfloxacin, novobiocin, fosfomycin	EmrAB (18)<br>thiolactomycin
E. cloacae	AcrA-dependent (20)<br>Piperacillin, cefuroxime, ceftazidime, cefotaxime, aztreonam, oxacillin, tobramycin, gentamicin, amikacin, tetracycline, erythromycin, tigecycline, telithromycin, clindamycin, ciprofloxacin, nalidixic acid, norfloxacin, trimethoprim-sulfamethoxazole, chloramphenicol, imipenem, meropenem, fusidic acid, novobiocin, linezolid, rifampin	AcrAB (1, 21)<br>cefepime, norfloxacin, levofloxacin, ciprofloxacin, moxifloxacin, tetracycline, tigecycline, tobramycin, erythromycin, chloramphenicol, colistin, trimethoprim-sulfamethoxazole, fusidic acid	AcrAD (1)<br>fusidic acid<br>AcrEF (1)<br>erythromycin<br>MdtABC (1)<br>fusidic acid
E. hormaechei	AcrAB (22)<br>Temocillin, cefepime,		

	tigecycline, ciprofloxacin, chloramphenicol
E. asburiae	AcrAB (23)<br>colistin

References

1. Guerin F, Lallement C, Isnard C, Dhalluin A, Cattoir V, Giard JC. Landscape of Resistance-Nodulation-Cell Division (RND)-Type Efflux Pumps in Enterobacter cloacae Complex. Antimicrob Agents Chemother. 2016;60(4):2373-82.
2. Nagano K, Nikaido H. Kinetic behavior of the major multidrug efflux pump AcrB of Escherichia coli. Proc Natl Acad Sci U S A. 2009;106(14):5854-8.
3. Lim SP, Nikaido H. Kinetic parameters of efflux of penicillins by the multidrug efflux transporter AcrAB-TolC of Escherichia coli. Antimicrob Agents Chemother. 2010;54(5):1800-6.
4. Li XZ, Plesiat P, Nikaido H. The challenge of efflux-mediated antibiotic resistance in Gram-negative bacteria. Clin Microbiol Rev. 2015;28(2):337-418.
5. Haeili M, Shoghi Y, Moghimi M, Arash G, Omrani M, Cirillo DM. Genomic features of in vitro selected mutants of Escherichia coli with decreased susceptibility to tigecycline. J Glob Antimicrob Resist. 2022.
6. Ma D, Cook DN, Alberti M, Pon NG, Nikaido H, Hearst JE. Molecular cloning and characterization of acrA and acrE genes of Escherichia coli. J Bacteriol. 1993;175(19):6299-313.
7. Sundaramoorthy NS, Sivasubramanian A, Nagarajan S. Simultaneous inhibition of MarR by salicylate and efflux pumps by curcumin sensitizes colistin resistant clinical isolates of Enterobacteriaceae. Microb Pathog. 2020;148:104445.
8. Chang TM, Lu PL, Li HH, Chang CY, Chen TC, Chang LL. Characterization of fluoroquinolone resistance mechanisms and their correlation with the degree of resistance to clinically used fluoroquinolones among Escherichia coli isolates. J Chemother. 2007;19(5):488-94.
9. Sulavik MC, Houseweart C, Cramer C, Jiwani N, Murgolo N, Greene J, et al. Antibiotic susceptibility profiles of Escherichia coli strains lacking multidrug efflux pump genes. Antimicrob Agents Chemother. 2001;45(4):1126-36.
10. Hirakawa H, Nishino K, Hirata T, Yamaguchi A. Comprehensive studies of drug resistance mediated by overexpression of response regulators of two-component signal transduction systems in Escherichia coli. J Bacteriol. 2003;185(6):1851-6.
11. Nishino K, Yamaguchi A. Analysis of a complete library of putative drug transporter genes in Escherichia coli. J Bacteriol. 2001;183(20):5803-12.
12. Elkins CA, Nikaido H. Substrate specificity of the RND-type multidrug efflux pumps AcrB and AcrD of Escherichia coli is determined predominantly by two large periplasmic loops. J Bacteriol. 2002;184(23):6490-8.
13. Nishino K, Yamada J, Hirakawa H, Hirata T, Yamaguchi A. Roles of TolC-dependent multidrug transporters of Escherichia coli in resistance to beta-lactams. Antimicrob Agents Chemother. 2003;47(9):3030-3.
14. Kawamura-Sato K, Shibayama K, Horii T, Iimuma Y, Arakawa Y, Ohta M. Role of multiple efflux pumps in Escherichia coli in indole expulsion. FEMS Microbiol Lett. 1999;179(2):345-52.
15. Elkins CA, Nikaido H. Chimeric analysis of AcrA function reveals the importance of its C-terminal domain in its interaction with the AcrB multidrug efflux pump. J Bacteriol. 2003;185(18):5349-56.
16. Nishino K, Yamaguchi A. EvgA of the two-component signal transduction system modulates production of the yhiUV multidrug transporter in Escherichia coli. J Bacteriol. 2002;184(8):2319-23.
17. Nagakubo S, Nishino K, Hirata T, Yamaguchi A. The putative response regulator BaeR stimulates multidrug resistance of Escherichia coli via a novel multidrug exporter system, MdtABC. J Bacteriol. 2002;184(15):4161-7.
18. Furukawa H, Tsay JT, Jackowski S, Takamura Y, Rock CO. Thiolactomycin resistance in Escherichia coli is associated with the multidrug resistance efflux pump encoded by emrAB. J Bacteriol. 1993;175(12):3723-9.
19. Kobayashi N, Nishino K, Yamaguchi A. Novel macrolide-specific ABC-type efflux transporter in Escherichia coli. J Bacteriol. 2001;183(19):5639-44.
20. Perez A, Canle D, Latasa C, Poza M, Beceiro A, Tomas Mdel M, et al. Cloning, nucleotide sequencing, and analysis of the AcrAB-TolC efflux pump of Enterobacter cloacae and determination of its involvement in antibiotic resistance in a clinical isolate. Antimicrob Agents Chemother. 2007;51(9):3247-53.

21. Liu L, Yu J, Tang M, Liu J. Mechanisms of Resistance in Clinical Isolates of Enterobacter cloacae that Are Less Susceptible to Cefepime than to Ceftazidime. Ann Clin Lab Sci. 2018;48(3):355-62.
22. Gravey F, Cattoir V, Ethuin F, Fabre L, Beyrouthy R, Bonnet R, et al. ramR Deletion in an Enterobacter hormaechei Isolate as a Consequence of Therapeutic Failure of Key Antibiotics in a Long-Term Hospitalized Patient. Antimicrob Agents Chemother. 2020;64(10).
23. Telke AA, Olaitan AO, Morand S, Rolain JM. soxRS induces colistin hetero-resistance in Enterobacter asburiae and Enterobacter cloacae by regulating the acrAB-tolC efflux pump. J Antimicrob Chemother. 2017;72(10):2715-21.
