# Supplementary material for: A comprehensive description of the TolC effect on the antimicrobial susceptibility profile in Enterobacter bugandensis
Source: Front Cell Infect Microbiol. 2022 Dec 9;12:1036933. doi: 10.3389/fcimb.2022.1036933 (PMC9780596; doi:10.3389/fcimb.2022.1036933)
Supplement: Supplementary file 2 [file DataSheet_2.pdf]

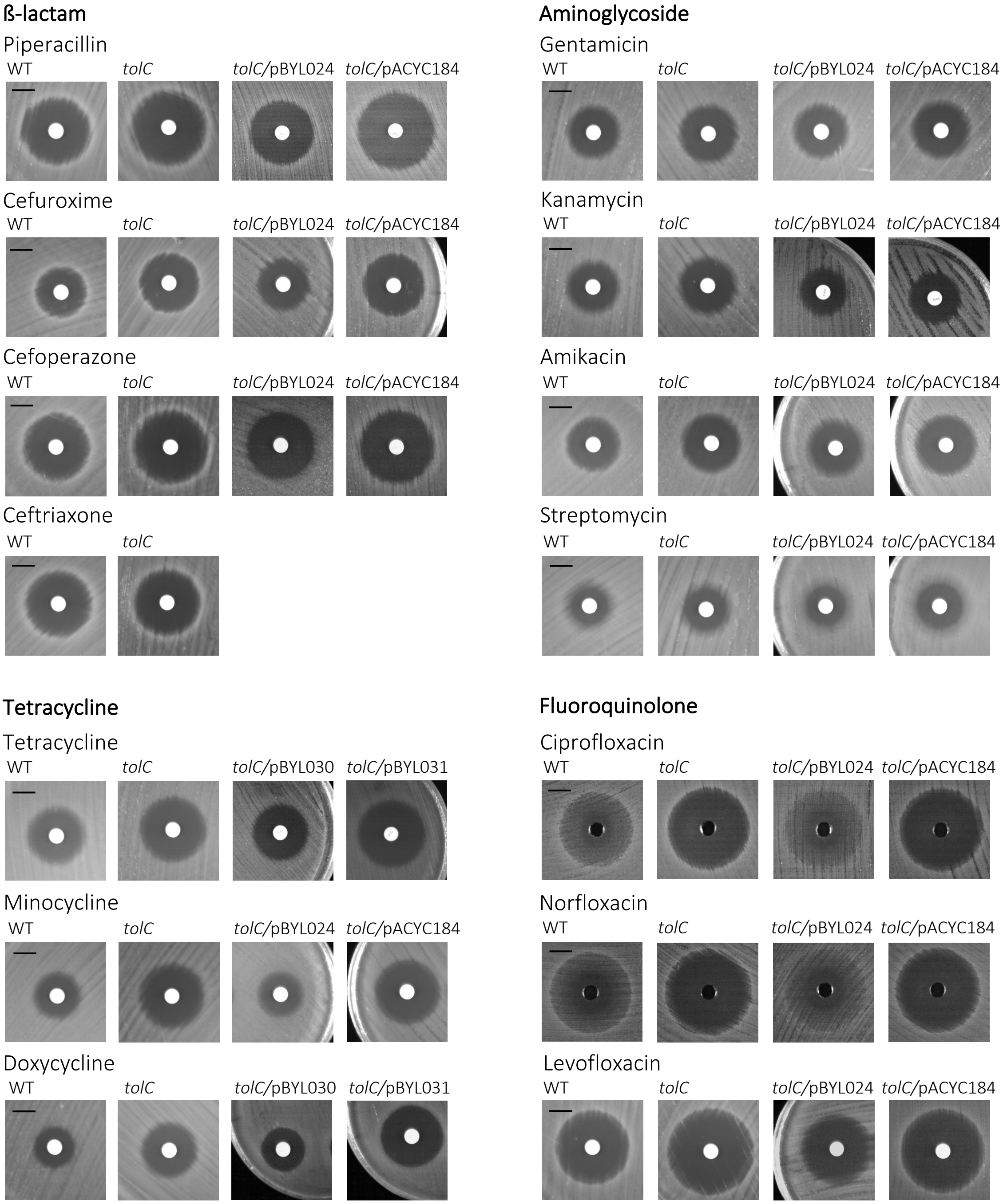


**Figure S1. The TolC effect on susceptibility to antibiotics belonging to families of ß-lactam, aminoglycoside, tetracycline, and fluoroquinolone in EBU45301.** The susceptibility of strain WT, *tolC*, *tolC*/complementation, and *tolC*/vector to piperacillin, cefuroxime, cefoperazone, ceftriaxone, gentamicin, kanamycin, amikacin, streptomycin, tetracycline, minocycline, doxycycline, and levofloxacin was tested using the disk-diffusion method (see materials and methods). The susceptibility of these strains to ciprofloxacin and norfloxacin was tested using the well-diffusion method (see materials and methods). For tetracycline and doxycycline, the used *tolC*/complementation and *tolC*/vector strains were *tolC*/pBYL030 and *tolC*/pBYL031, respectively. For the rest cases, the used *tolC*/complementation and *tolC*/vector strains were *tolC*/pBYL024 and *tolC*/pACYC184, respectively. The shown images are a representative of three independent experiments. All the black bars represent 10 mm.

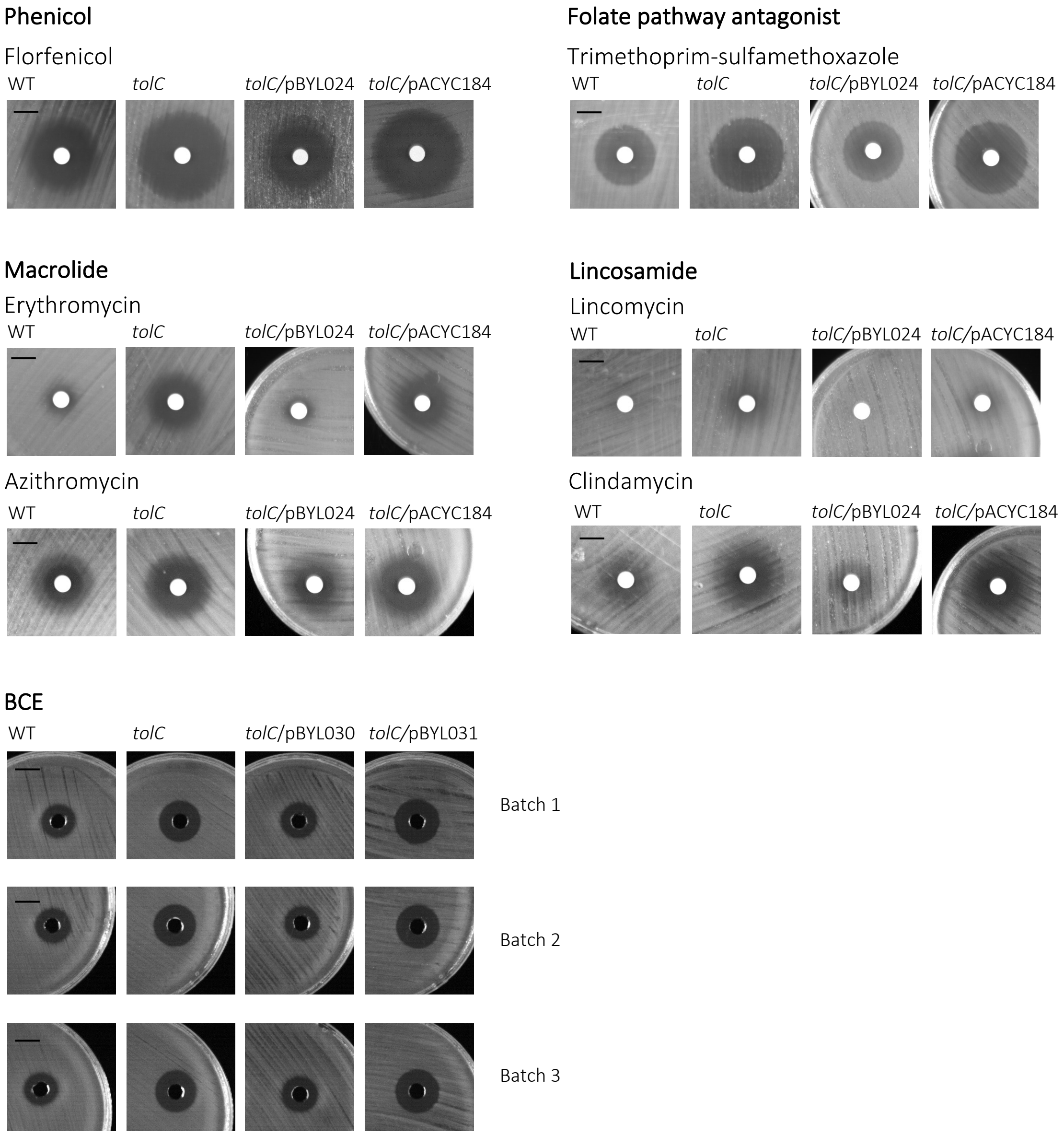


**Figure S2. The TolC effect on susceptibility to antibiotics belonging to families of phenicol, folate pathway antagonist, macrolide, lincosamide, and BCE in EBU45301.** The susceptibility of strain WT, *tolC*, *tolC*/complementation, and *tolC*/vector to florfenicol, trimethoprim-sulfamethoxazole, erythromycin, azithromycin, lincomycin, and clindamycin was tested using the disk-diffusion method. The susceptibility of these strains to BCE was tested using the well-diffusion method. For BCE, the used *tolC*/complementation and *tolC*/vector strains were *tolC*/pBYL030 and *tolC*/pBYL031, respectively. For the rest cases, the used *tolC*/complementation and *tolC*/vector strains were *tolC*/pBYL024 and *tolC*/pACYC184, respectively. The shown images are a representative of three independent experiments. All the black bars represent 10 mm.

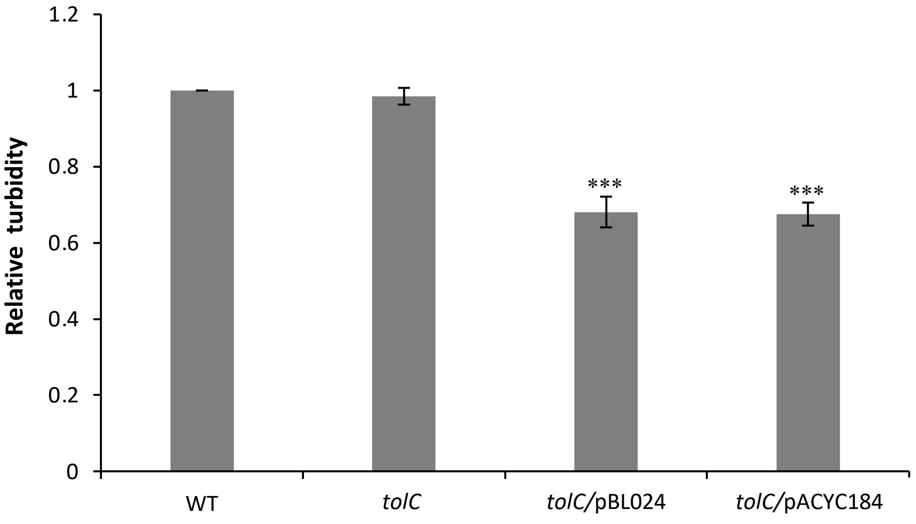


**Figure S3. A chart showing the relative turbidity of strains of WT, *tolC*, *tolC*/pBYL024, and *tolC*/pACYC184.** For each strain, the inoculum with a cell density of $5x10^5$ CFU/mL was prepared using the cation-adjusted Mueller-Hinton broth (CAMHB). The inoculum was grown in a 96-well microtiter plate at 37°C for 18 hours, and then its turbidity was measured as the $OD_{600}$ value. The turbidity of WT was normalized to 1, and that of the other strains was calculated as the value relative to 1. The relative turbidity for each strain was determined as the average ± SD of three independent experiments. The comparative analyses of data were performed using student's *t* tests. ***, $p \leq 0.001$ *versus* the turbidity of WT.

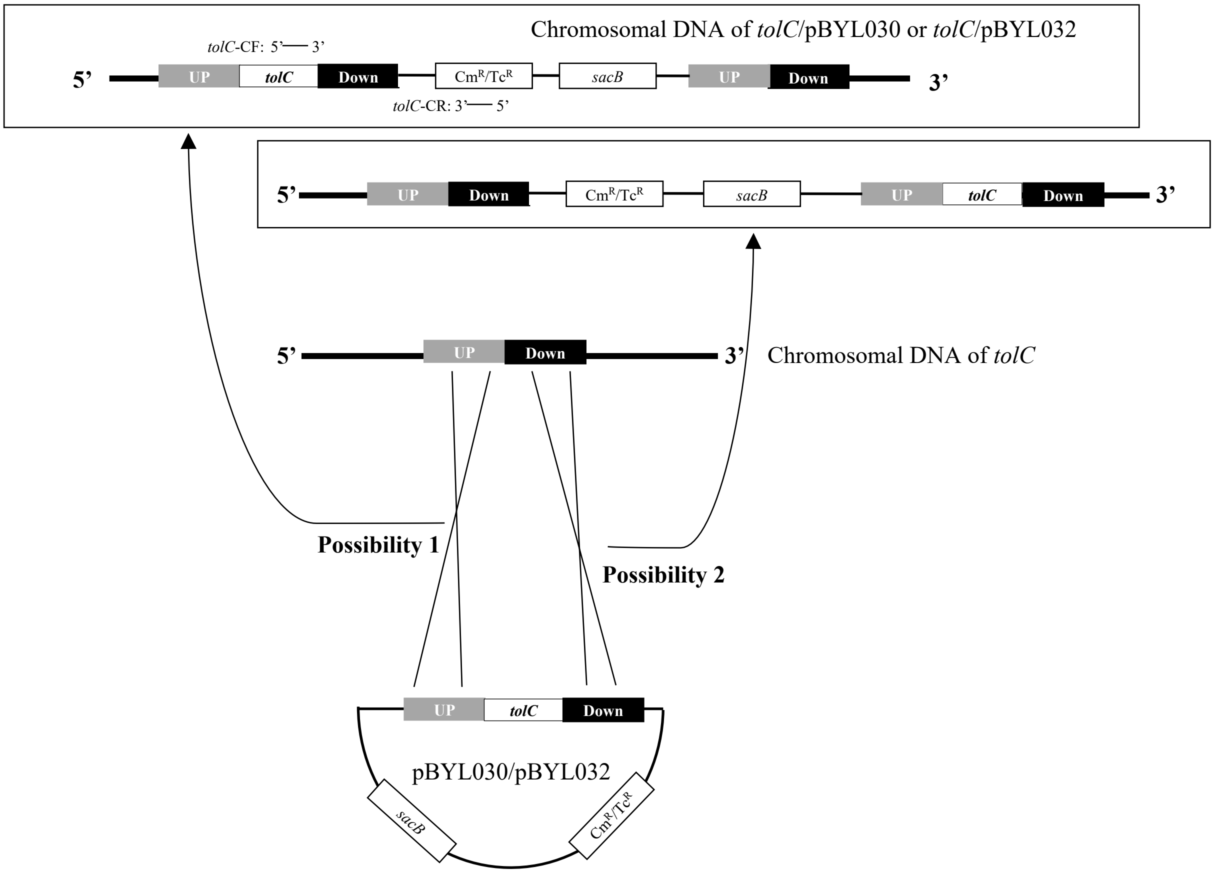


**Figure S4. The schematic showing the construction of strain *tolC*/pBYL030 and *tolC*/pBYL032.** The suicide plasmid pBYL030/pBYL032 was introduced into the *tolC* mutant and integrated in its chromosome via homologous recombination. The recombination can occur either via the "UP" homologues (shown as grey bars) or the "Down" homologues (shown as black bars), which causes different chromosomal arrangements of the DNA sequences after integration, as shown in the boxes for route "Possibility 1" and route "Possibility 2". To assure that the *tolC*/pBYL030 or *tolC*/pBYL032 isolates used for the antimicrobial susceptibility tests possess the same chromosomal DNA arrangement, we uniformly selected the ones resulted from recombination of "Possibility 1" as the complementing strain *tolC*/pBYL030 or *tolC*/pBYL032, which could be verified by PCR tests using the forward primer located within the *tolC* sequence and the reverse primer located within the $Cm^R$ or $Tc^R$ cassette.

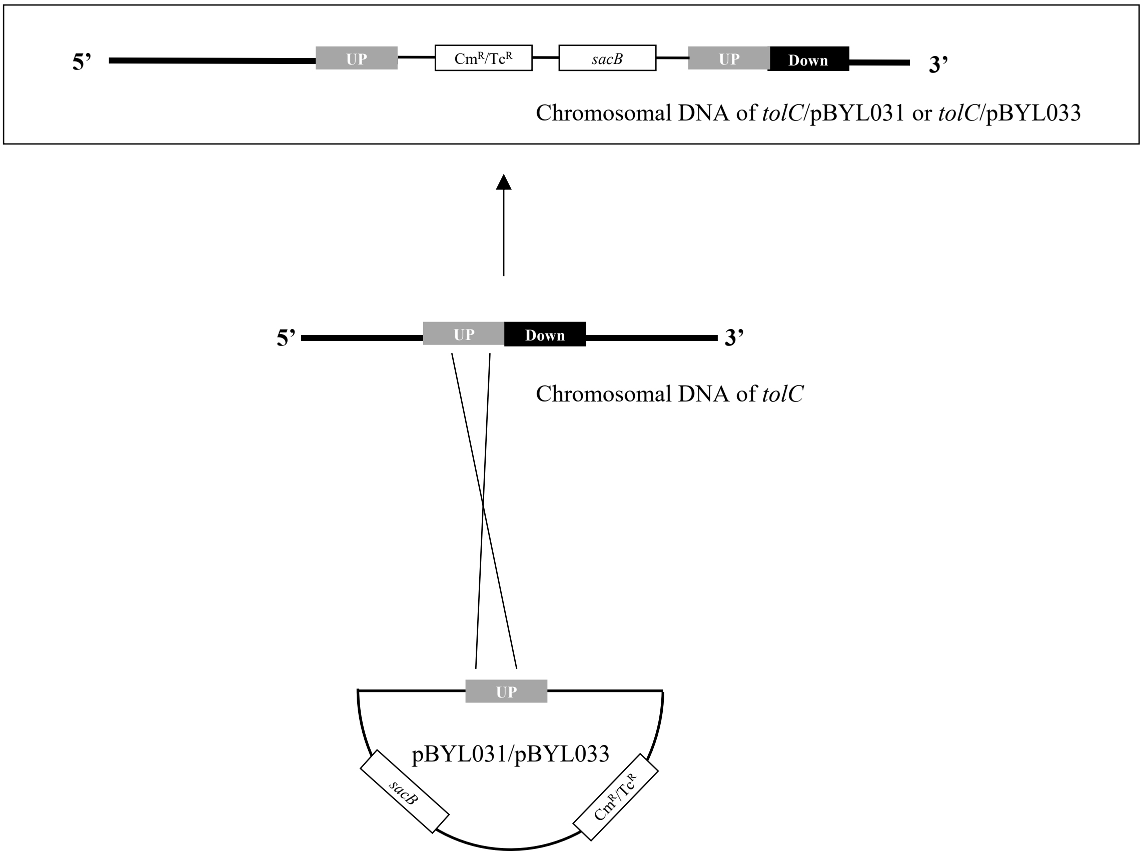


**Figure S5. The schematic showing the construction of strain *tolC*/pBYL031 and *tolC*/pBYL033.** The suicide plasmid pBYL031/pBYL033 was introduced into the *tolC* mutant and integrated in its chromosome via the homologous recombination occurred through the "UP" homologues (shown as grey bars). The chromosomal DNA arrangement of the resulted strain *tolC*/pBYL031 or *tolC*/pBYL033 was shown in the box above.
